# Supplementary material for: Effects of SLC45A2 and GPNMB on Melanin Deposition Based on Transcriptome Sequencing in Chicken Feather Follicles
Source: Animals (Basel). 2023 Aug 12;13(16):2608. doi: 10.3390/ani13162608 (PMC10451703; doi:10.3390/ani13162608)
Supplement: Supplementary file 1 [file animals-13-02608-s001.zip › Figure S1.pdf]

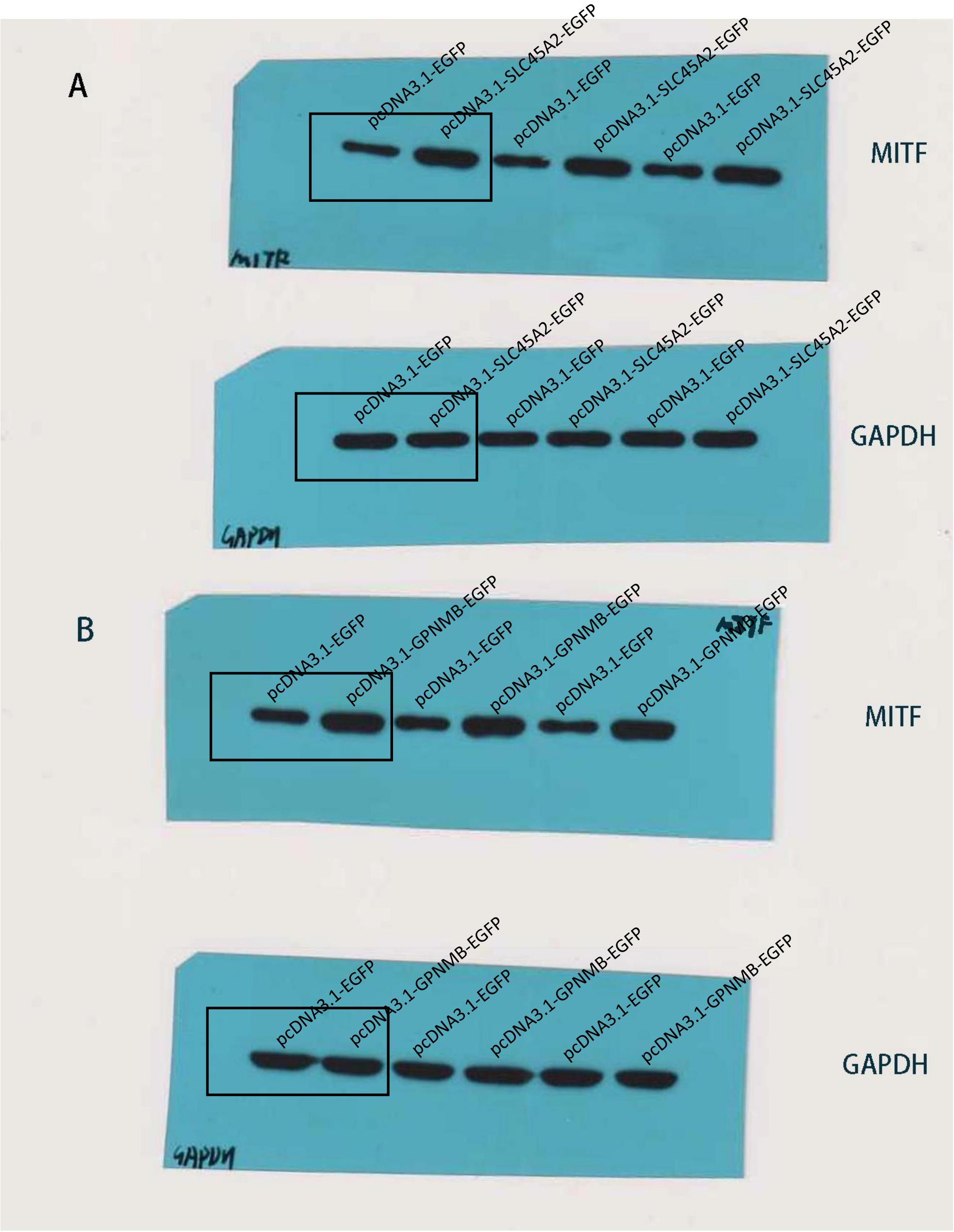

Figure S1: Full Western Blots representing images used in Figure 8F and G. (**A**) The expression of MITF and GAPDH proteins was detected after overexpression of *SLC45A2* in chicken melanocytes. (**B**) The expression of MITF and GAPDH proteins was detected after overexpression of *GPNMB* in chicken melanocytes. There were 3 replicates in each group.
