# Supplementary material for: Effects of SLC45A2 and GPNMB on Melanin Deposition Based on Transcriptome Sequencing in Chicken Feather Follicles
Source: Animals (Basel). 2023 Aug 12;13(16):2608. doi: 10.3390/ani13162608 (PMC10451703; doi:10.3390/ani13162608)
Supplement: Supplementary file 1 [file animals-13-02608-s001.zip › Figure S2.pdf]

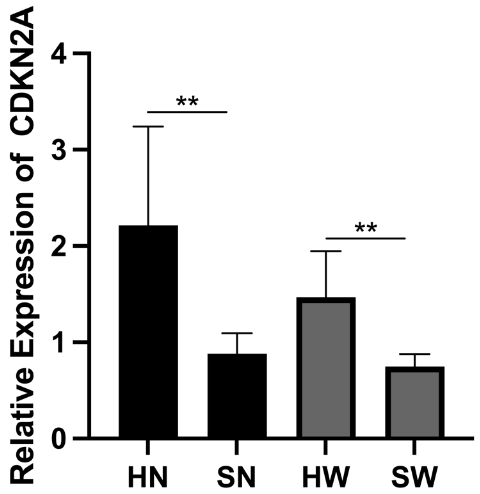

Figure S2: Differences in the relative expression of CDKN2A in the two comparison groups. HN group(sub-Columbian plumage nuchal follicle tissue) vs. SN group (sliver plumage nuchal follicle tissue), HW group (sub-Columbian plumage wing follicle tissue) vs. SW group (white plumage wing follicle tissue). Each sample had three biological replicates and three technical replicates.

** indicates $p < 0.01$
