# Supplementary material for: Effects of SLC45A2 and GPNMB on Melanin Deposition Based on Transcriptome Sequencing in Chicken Feather Follicles
Source: Animals (Basel). 2023 Aug 12;13(16):2608. doi: 10.3390/ani13162608 (PMC10451703; doi:10.3390/ani13162608)
Supplement: Supplementary file 1 [file animals-13-02608-s001.zip › Table S1.pdf]

Table S1. List of overexpression vector construction and and qPCR primers.

Gene	Primer	Sequence(5'-3')	Size (bp)
**A. Specific primers used for overexpression vector construction of *SLC45A2* and *GPNMB***			
SLC45A2	Forward	CTAGCGTTTAAACTTAAGCTTATGACCCTAACGGAACAGCAC	1679
	Reverse	GCACCGTCGACTGCAGAATTCCCCCACATATCGAACACAGAA	
GPNMB	Forward	GCTGGCTAGCGTTTAAACTTAAGCTTATGTGCGGGGCTCACCGC	1724
	Reverse	GCACCGTCGACTGCAGAATTCAACAATGCCTGGTTTGCTTTTC	
**B. Specific primers used for qRT-PCR**			
COL1A1	Forward	CGGGCTGACGTACAACGATA	145
	Reverse	GCACTCTCCGAAGGGGATTT	
AQP1	Forward	TTCCCAGTGTCAGTCAACGG	81
	Reverse	GGTCGCGATGGATAACCCAA	
CYR61	Forward	AGTACTGTGGGTCTTGCGTG	163
	Reverse	TAAGCTTCGTTTGCATGCGG	
RGN	Forward	GACTGGTCGCTGGATCACAA	257
	Reverse	ACAGGAAGCTTCACCGTCTG	
KRT23	Forward	AAAGCATCTTCCTGGGTGGG	280
	Reverse	AAGTCTTCGGACGCCATGTT	
SLC45A2	Forward	GCACTGGGTTGATTGGCTTG	147
	Reverse	GCTGCAGATTCAGGTCCTCTT	
GPNMB	Forward	CCACACCTCTTCACTCGTCC	122
	Reverse	CCGCTCTGAAATGCCACAAC	
INAFM2	Forward	CACTTACACCGGGGACAAGA	126
	Reverse	TAGTAGACGGCGAGGACGAT	
NFKBIE	Forward	CTCACCTACGTCTCGGAGGA	116
	Reverse	TGGATCTCCAGCAGCTCTCT	
TYR	Forward	TCATGGGGTTCAACTGTGGG	221
	Reverse	TCTGAACATGGGGTTGGAGC	
MLPH	Forward	CCCGCTTCAAGAGGTTTGGA	237
	Reverse	AATCTGGGAAGGATCTGCTGT	
MITF	Forward	CTGTGCTGGACAACTGCAAC	267
	Reverse	TCCGCCTGCTACTCGTTTTT	
CDKN2A	Forward	CTCTGTCCTTCTCGCTGCTC	107
	Reverse	GTGCCTCCTTCTCAGAACCC	
**C. Primers of the internal control gene**			
GAPDH	Forward	GAACATCATCCCAGCGTCCA	210
	Reverse	CGGCAGGTCAGGTCAACAAC	
