# Supplementary material for: Effects of SLC45A2 and GPNMB on Melanin Deposition Based on Transcriptome Sequencing in Chicken Feather Follicles
Source: Animals (Basel). 2023 Aug 12;13(16):2608. doi: 10.3390/ani13162608 (PMC10451703; doi:10.3390/ani13162608)
Supplement: Supplementary file 1 [file animals-13-02608-s001.zip › Table S2.pdf]

Table S2. Statistics of quality control and reads mapping of sequencing date.

Sample	Clean reads/ Mb	Mapped Reads/ Mb	Uniquely mapped reads/ Mb	Q20(%)	Q30 (%)	GC (%)
HW1	25.52	44.96	40.73	97.94	94.58	55.35
HW2	27.07	47.85	43.19	97.81	94.32	54.62
HW3	26.77	47.37	42.84	97.81	94.34	54.73
HN1	26.17	45.70	43.15	97.64	94.06	53.38
HN2	27.20	47.76	45.72	97.50	93.72	53.00
HN3	25.90	45.73	43.35	97.65	94.01	53.31
SW1	25.08	45.84	38.66	97.51	93.33	54.85
SW2	24.79	45.28	39.29	97.32	92.92	54.05
SW3	27.74	50.56	43.84	97.39	93.07	54.36
SN1	29.76	55.06	49.33	97.30	92.89	52.83
SN2	29.05	53.72	47.13	97.27	92.87	52.83
SN3	26.81	49.41	44.18	97.31	92.92	52.63
YW1	26.97	48.38	43.63	98.01	94.63	55.17
YW2	24.98	44.80	40.03	97.89	94.47	54.76
YW3	25.86	46.37	41.31	98.14	94.94	55.37
YN1	27.41	49.12	45.77	98.04	94.71	54.11
YN2	25.15	45.16	42.68	97.78	94.27	53.43
YN3	24.55	43.59	41.29	97.88	94.42	53.41

Note: Sample ID: Q20(%): percentage of bases with mass greater than or equal to 20 of the total number of bases; Q30(%): percentage of bases with mass greater than or equal to 30 of the total number of bases; GC (%): the percentage of G and C type bases in the total number of bases.
