# Supplementary material for: Effects of SLC45A2 and GPNMB on Melanin Deposition Based on Transcriptome Sequencing in Chicken Feather Follicles
Source: Animals (Basel). 2023 Aug 12;13(16):2608. doi: 10.3390/ani13162608 (PMC10451703; doi:10.3390/ani13162608)
Supplement: Supplementary file 1 [file animals-13-02608-s001.zip › Table S6.pdf]

Table S6. DEGs in the Melanogenesis pathway.

Gene Name	NR Annotation	BC vs HC			BQ vs_HQ1_HQ2_HQ3		
		FDR	log2FC	Regulated (FDR<0.05 FC≧1.5)	FDR	log2FC	Regulated (FDR<0.05 FC≧1.5)
ADCY3	adenylate cyclase type 3 isoform X2	0.001068448	0.722777894	up	3.20E-23	1.797203611	up
ADCY8	adenylate cyclase type 8 isoform X1	0.022645924	-0.943088984	down	1.71E-07	-2.099001904	down
WNT11	protein Wnt-11 precursor	3.41E-30	-1.728257367	down	8.44E-34	-1.657612256	down
FZD2	frizzled-2 precursor	4.35E-07	0.851122842	up	4.76E-17	1.415951835	up
FZD1	frizzled-1 precursor	4.63E-24	1.129167846	up	7.18E-22	1.002926166	up
FZD7	frizzled-7 precursor	6.94E-18	1.096783635	up	4.83E-12	1.069554095	up
FZD9	frizzled-9 precursor	6.43E-17	1.189729697	up	1.06E-15	1.055256184	up
